# Sport and health science: interdisciplinary approaches to modern challenges

**DOI:** 10.1093/bmb/ldaf007

**Published:** 2025-06-22

**Authors:** Johnny Padulo

**Affiliations:** Department of Biomedical Sciences for Health, Università degli Studi di Milano, 20133 Milan, Italy

**Keywords:** health science, exercise physiology, lifestyle, physical activity, public health

## Abstract

**Background:**

Sport and health science are undergoing a transformative shift driven by interdisciplinary approaches, technological innovations, and data-driven strategies.

**Sources of data:**

This invited editorial explores key advancements in precision athletic monitoring, holistic well-being, population health initiatives, and innovative training and rehabilitation techniques. The integration of wearable technology, real-time analytics, and psychological interventions enables more personalized and effective strategies to optimize performance and promoting overall health. Additionally, the role of physical activity in mitigating lifestyle-related diseases underscores the importance of tailored public health initiatives.

**Areas of agreement:**

Current consensus in sport and health science highlights that interdisciplinary collaboration—blending physiology, psychology, nutrition, and data analytics—significantly improves both athletic performance and public health outcomes. This progress is driven by precision monitoring technologies, holistic well-being strategies, innovative training methods, and a shared commitment to ethical standards ensuring equitable, responsible application of new health, and performance innovations.

**Areas of controversy:**

As the field progresses, ethical considerations regarding data privacy, accessibility, and equitable application of emerging technologies remain central.

**Growing points:**

Future directions include leveraging artificial intelligence, machine learning, and big data to refine personalized interventions, ensuring that both athletic and public health advancements are sustainable and inclusive.

**Areas timely for developing research:**

Emerging research in sport and health science focuses on harnessing artificial intelligence, machine learning, and big data to develop predictive models and personalized interventions, while tailoring physical activity programmes to diverse population needs based on age, gender, socioeconomic, and cultural factors. Simultaneously, priorities include advancing psychological and mindfulness-based strategies in athletic care, integrating cutting-edge rehabilitation technologies, promoting inclusive public health frameworks for ageing and chronically ill populations, and establishing ethical guidelines for the responsible use of innovative performance and health technologies.

## Introduction

The landscape of sport and health science is evolving rapidly, as innovative technologies, novel research methodologies, and interdisciplinary approaches redefine our understanding of human performance and wellness. In today’s interconnected world, athletic performance, public health, and rehabilitation strategies are no longer viewed in isolation [1]. Instead, these areas are increasingly examined through the integration physiology, biomechanics, psychology, nutrition, and data analytics [2]. This synthesis has allowed researchers and practitioners to develop more comprehensive, personalized strategies to improve physical performance and promote overall well-being. As we address modern challenges—from optimizing athlete training regimens to curbing lifestyle-related diseases—the need for collaboration across disciplines becomes ever more apparent.

In this invited editorial, we explore the emerging themes in sport and health science, highlighting the technological advancements and scientific innovations that have propelled the field into a new era. Emphasis is placed on the importance of precision monitoring, holistic well-being, population health initiatives, and novel training methods—all underpinned by ethical considerations and future directions that promise to shape the field in years to come.

### The era of precision in athletic monitoring

One of the most remarkable advancements in sport and health science is the adoption of precision monitoring [2]. Wearable technology [3,4], combined with real-time data analytics, has revolutionized the way in which athletes and health professionals track performance, recovery, and overall health [5]. These devices not only measure traditional metrics such as heart rate, distance, and speed, but they also provide insights into more complex physiological parameters [6]. For instance, the latest wearables are also continuous glucose monitors, sleep trackers, and stress indicators [7].

The ability to collect and analyse such rich datasets allows coaches and medical professionals to design individualized training programmes that optimize performance while minimizing the risk of injury [8]. Moreover, this data-driven approach has enabled to identify early warning signs of overtraining and other adverse conditions, paving the way for timely interventions. As we move toward personalized medicine [9], the integration of advanced analytics with traditional training methods represents a paradigm shift in athletic care and performance optimization.

### Holistic approaches to well-being

Optimal performance and health extend beyond mere physical conditioning. Modern athletes’ data allow to consider athletes as much a mental and emotional being as a physical one. Holistic approaches to well-being acknowledge that psychological resilience, stress management, and social factors play crucial roles in both athletic success and overall health outcomes. Sports psychologists now work alongside physiologists, nutritionists, and trainers to ensure that every facet of an athlete’s well-being is addressed.

Interventions that incorporate mindfulness, cognitive behavioural strategies, and even creative therapies are increasingly being integrated into training and rehabilitation protocols [9]. These methods not only help athletes cope with the pressures of competition but also contribute to long-term mental health. The benefits of such integrated approaches are evident not only in enhanced performance metrics but also in reduced incidences of burnout and mental health challenges among athletes and the broader population [10].

### Population health and the role of physical activity

The benefits of physical activity extend far beyond the realm of elite sports. As chronic non-communicable diseases such as obesity, cardiovascular disorders, and diabetes become more prevalent, public health initiatives are placing a renewed emphasis on physical activity as a cornerstone of disease prevention and management. Regular exercise can significantly reduce the risk of developing these conditions, improve mental health, and enhance quality of life [11].

However, the implementation of physical activity programmes must consider the diverse needs of various demographic groups. Age, gender, socioeconomic status, and cultural background can all influence how individuals engage with exercise. For example, while high-intensity training may be suitable for younger, competitive athletes, more moderate, sustainable forms of activity are recommended for older adults or those with pre-existing health conditions. By developing population-specific guidelines and interventions, public health officials can more effectively promote physical activity across diverse communities.

### Innovative training and rehabilitation techniques

The field of sport and health science continues to push the envelope with innovative training and rehabilitation techniques [9,12] that are transforming how athletes and patients recover and achieve peak performance. High-intensity interval training, e.g. has gained widespread popularity for its efficiency in improving cardiovascular health and metabolic function [13]. Its success lies in the ability to provide significant health benefits in a shorter period compared to traditional endurance training.

At the same time, advancements in rehabilitation protocols—ranging from novel strength training methods to the integration of technology such as virtual reality and robotics—are revolutionizing the recovery process for injured athletes and patients alike [14]. These methods not only accelerate physical recovery but also aid in restoring confidence and functional independence. The cross-pollination of ideas between sport science and clinical rehabilitation has resulted in therapeutic strategies that are both evidence-based and tailored to individual needs, offering hope for improved outcomes in a range of conditions.

### Ethical considerations and future directions

As technological and methodological innovations proliferate in sport and health science, ethical considerations have taken centre stage. Issues surrounding data privacy, equitable access to advanced technologies, and the ethical use of performance-enhancing interventions are now critical areas of discussion. Researchers and practitioners must navigate these challenges with a commitment to transparency and fairness, ensuring that the benefits of scientific advancements are accessible to all, rather than a select few.

Looking ahead, the future of sport and health science is poised to be shaped by further interdisciplinary collaborations. The integration of artificial intelligence, machine learning, and big data analytics shall likely refine our understanding of human performance and health. Such technologies will enable more sophisticated predictive models and personalized interventions, ushering in a new era of preventive and personalized medicine.

Moreover, the global challenges of an ageing population and rising chronic disease prevalence necessitate a shift toward more inclusive and sustainable approaches to physical activity and health promotion. By leveraging the collective expertise from multiple disciplines, the field can develop innovative solutions that not only enhance athletic performance but also improve public health outcomes.

## Conclusion

The evolution of sport and health science is a testament to the power of interdisciplinary collaboration. By embracing precision monitoring, holistic well-being, population-specific strategies, and innovative training techniques, researchers, and practitioners are breaking new ground to understand and improve human performance. As we look to the future, ethical considerations and technological advancements will continue to shape the field, ensuring that progress is both equitable and sustainable.

This invited editorial has provided a broad overview of the transformative trends in sport and health science. For those interested in deeper dives into specific research areas, a dedicated Further Reading section is provided below, where specialized topics are discussed in greater detail.

## Data Availability

The data that support the findings of this study are available from the corresponding author, upon reasonable request.
